# Metanoia:—A Plea for the Insane

**Published:** 1861-04

**Authors:** Henry McCormac

**Affiliations:** Visiting Physician to the Belfast District Hospital for the Insane Poor, and Consulting Physician to the Belfast General Hospital.


### Art. IX.—METANOIA.

### A PLEA FOR THE INSANE.

By Henry McCormac, M.D., Visiting Physician to the Belfast District Hospital for the Insane Poor, and Consulting Physician to the Belfast General Hospital.

The soul is beset, or liable to be beset, by different influences, which raise or depress while they occupy it, from the highest to the lowest level, from perfect sanity of heart and mind to perfect insanity, and from perfect goodness, and purity, and truth, to the utterest impurity and untruthfulness. The souls of the insane, however deranged, perverted, or destroyed, are governed by the same laws as the souls of the sane. If we do not understand the psychology of health, we shall never understand the psychology of disease. For there is no exact line of severment between sanity and insanity. There are perhaps as many mutations in insane as in sane life. Yet the insane man is not entirely insane, nor is the sane man always entirely sane. It is the preponderance, in amount and degree, of insanity over sanity that constitutes disease.

The insane man perceives, but his perceptions are distorted, imperfect, confused. His attention, and also, his association of ideas, are similarly characterized by disorder and decay. Yet are they demonstrative of eternal laws, divine realities, subsistent in every breast. As in dreaming, somnambulism, delirium, reason's rule has ceased, the laws of the succession of thought or suggestion still prove operative, but all strict control is at an end.

Corporeal disease, nerve deficiency, may lead to insanity, and are often associated with insanity, oftener still with idiotcy, but corporeal disease is not insanity. It is not true that in the morbid states of thought, which we term insanity, a change in the material organization is necessarily involved, and simply, because thought is not the product of organization. Disordered sensation, hallucination also, which is a form of disordered sensation, may lead to insanity, but, as in the famous instance of Pascal, may subsist quite apart. The brain, neither the white part nor the grey, is not mind, does not discharge any one mental function, is of no intellectual capacity or potentiality whatever. So far as mind is concerned, the brain is simply a large ganglion, and associated with the spinal marrow or nerves, generally, a congeries of organs for the generation and propagation of nerve-force, the vehicle of impressions from the sense-organs, and of conveying to those organs the soul's volitions. It is just as reasonable to ascribe consciousness to brain fibre as it would be to ascribe consciousness to a turnip or a stone.

We may assume, if we like, that life precedes consciousness. Doubtless, however, the mental, plus the bodily consciousness, is generated together. Outward objects, as we know them, and, irrespective of the outward factor, which, the soul's receptivity being assumed, generates them, are but forms of the soul's consciousness. There is a concrete ego. The extended living frame, indeed all nature, as we know it, subsists in the consciousness. But the living frame, from its ceaseless contiguity, differing thus from other outward objects, is continually present as an objective consciousness. A subjective consciousness, indeed, ends in self, whereas the objective consciousness assumes an outward factor, conterminous with, and inclusive of the sentient nerves. Whosoever does not admit the foregoing, does not admit the first elements, the A B C of psychology. The soul-mind, then, and the body-mind, so to speak, as respects the plane of consciousness, are one. But the different states of the object-mind precede and give rise, in the first instance, to the different states of the subject-mind itself, which then gains the capacity of thus continuing and propagating itself, in consciousness, for ever.

Thus, then, there is, there can be, no conscious existence or subsistence out of the plane of the conscious mind itself, and consequently, the notion that insanity is merely a cerebral disease, a disorder of the delicate vesicular neurine, or nerve-substance, is only fit for that limbo to which a Milton consigns things shapeless, fantastic, unreal. The whole hypothesis is at issue with the deliverances of consciousness as a truth-organ. Now, the entire formation of character, of all individuality, in the sane as in the insane, resides in fixity of impression. It is God's law as impressed on nature and the soul of man, and is alike true, whether in regard of sensation, emotion, or thought. Without fixity of impression one man would not differ from another man, nay, would not be himself from one interval of time to another. Fixity of impression, in its degree, is a thing so important, so all-essential, that nature, by which I would understand the provident working of God, has employed every desirable, and indeed possible means to realize it. Without fixity of impression there could be no education, no training, because there would be no real, at least no lasting receptivity. There would be no science nor art, nor any genius or skill. So, then, congruous with the laws of consciousness, it is fixity of impression, the appliances being conformable, that characterizes, indeed creates, sanity in the sane, and in the insane insanity.

The antithesis of fixity of impression is mutability of impression. Without mutability, in its degree, fixity would be useless, without fixity mutability would be similarly useless. There must be a healthy mutability, a healthy fixity, neither in excess, neither unduly deficient. There may be an insane mutability as well as an insane fixity, a sane fixity, a sane mutability. The healthy soul includes both healthily.

It must not be supposed that all insane persons become insane in the sense of lapsing from sanity into insanity. There is such a thing as an insane development, being insane, because in strictness there never was perfect sanity. The paucity, the sparseness of intelligence which multitudes of insane persons display, idiotcy in all its grades, weak-mindedness in all its grades, can be explained on no other principle. Here, then, the faculties are few in number, the intelligence is limited. The faculties are poorly, and not only poorly but insanely exercised. Of the many forms of insanity, and insanity is indeed various, this of undeveloped, ill-developed intelligence, is perhaps the most difficult to deal with.

For the most part insanity is of slow growth. Many insane persons remain long years unchanged, do not become better, neither do they grow worse. On the other hand, while some regain their normal intelligence, others lapse into utter imbecility. If serious bodily disorder, if paralysis, local or general, if epilepsy coexist, the tendency, other things being equal, is evil. I do not find a lightening up or restoration of the intelligence before death. In fact, I do not remember an instance of it among the insane persons whom I have seen dying or about to die. They die in general as they have lived, insane.

Idiotcy or dementia subsists from birth, or it may supervene. In the former case, the nerve-structure, though deficient, may be otherwise healthy. When there is deficiency in the nerve-apparatus, the great ganglion which we term the brain, the functions dependent on this apparatus will be deficient, the organs of relation will be at fault But this nerve-deficiency may ensue later in life, owing to cerebral structural disease. It may arise also from sudden and violent shocks, fright, terror, surprise. At other times the faculties languish, vegetate, and decay, concurrently with general prostration and decay, but apart from any appreciable nervous organic change.

Insanity, unless when early removed, tends to chronicity, and may subsist for years. And yet, even when removed, it may, and often does recur. Anything that turns the attention, that incites to wholesome instead of unwholesome thought, to control, instead of want of control, to sound in place of morbid will, is of service. And thus it is, other circumstances proving favourable, that the prospect of recovery is so much greater among strangers than among friends, abroad than at home. Persons otherwise insane, on being visited by friends or strangers, will often place such constraint on themselves as to seem, and indeed for the time to be, quite sane. The will, in effect, is the man, and if we can but healthily control it, we have the Open Sesame which happily is to conduct us into the treasure-house of reason itself. If the return from insanity to sanity, as I firmly believe and maintain, be realized by the culture of the will, it sets aside, as irreconcileable with fact and with truth, the doctrine that the one and only genetic source of insanity is organic, structural, cerebral change.

Delirium differs from insanity in that it has a corporeal origin, as evidenced in febrile disorders, during inflammation, the accidents of parturition, hysteria, and as owing to loss of blood. In the crises of excessive excitement, violent passion, epidemies of fanaticism, deranging body and soul, the mental functions are perturbed to that degree that insanity itself is a not unfrequent result. In the delirium induced by toxic drinks, *i.e.*, strong drinks in toxic doses, the abuse of narcotics, the nerves, both at the centre and the periphery, are subjected to false impressions. Hallucination from disordered nerve-function, as I have said, may subsist in insanity, is even capable of inducing it. But, as noted instances prove, the case of the Berlin bookseller Nicolai, for example, hallucination may co-exist with otherwise perfect mental health. Irregular nerve-function, irregular reflex-function, leading occasionally to delirium and madness, may be seen in somnambulism, ecstasy, and the states induced by what in America is named Spiritualism. Insanity from terror, bereavement, shocks of surprise, may, and does ensue without appreciable structural change, and, along with instances of ordinary chronic insanity, contradict and set aside the notion of the exclusive corporeal origin of psychical derangement.

If, then, we discard this error as to the cerebral origin of insanity, we must as a corollary dismiss likewise the equally erroneous doctrine of hereditary insanity. But the condition of brain or of nerve-fibre, productive, as asserted, of insanity, it will be said is hereditary. Yes, but thought is not a nerve-function at all, does not depend on phosphorus, as modern empirics have it, is not of molecular origin, whether in the sane or the insane, con sequently insanity is not thus, cannot be, hereditary. It would be strange indeed if insane people had not sometimes insane offspring, since we find it so even with the sane. Doubtless the mischievous influence of imitation, example, of moral infection, so to speak, will do its work, but then this is not the hereditary influence contended for. I do not mean to say that impairment of nerve-structure may not be derived, as numberless instances of congenital idiotcy from hereditary influences prove. But the deficiency of nerve-action which arises from deficient nerve-structure is quite a distinct thing from the mental derangement which may, and does ensue, without any cerebral deficiency, structural or functional, whatever. For the nerve-structures discharge the functions of organs of relation and innervation, and have nothing to do with thought, which is not a nerve-function at all. The doctrine of hereditary insanity is a mischievous error, fraught, like all the other fruits of our ignorance and misconception, with evil and bale.

The mortality during insanity varies, but is at all times considerable. For the health suffers from detention, gloom, cells ill-ventilated, mental strife, constraint, coupled with the ills incident to our common humanity. The insane rally but indifferently from attacks of disease, whether chronic or acute. Little co-operation is too often to be expected from them even in respect of the best concerted measures for their relief. If we assume the total number of lunatics, idiots, and demented persons in Britain, Ireland, and their dependencies as 50,000, and if we further assume the annual mortality as 10 per cent., we find that the accession of insane persons needful to maintain this amount is about 5000 every year. Such are the disastrous results, not indeed of hereditary influence or organic disease, so much as of defective training, weakened self-restraint, utter neglect.

Everything connected with a healthy will, very especially the faculties of perception and sustained attention, is excessively impaired in the insane. This necessarily follows from the weakening and impairment of the faculty of self-control itself, so needful to the exercise of healthy thought. The power of attention differs in the insane, as it differs in the sanely-minded themselves, but is lessened in all. Few things indeed are more remarkable in contemplating the insane than the indifference which they manifest under circumstances which arrest the attention of persons in sane life.

There is one thing which, as it seems to me, lies at the root of the whole question of insanity, its pathogenesis and treatment alike. I mean the manner of the consciousness in the insane. This is a matter of vastly greater moment than are any considerations relative to real or fancied mutations in what some are pleased to term the vesicular neurine of the brain, the plus, or the minus, real or fancied, of nerve-phosphorus. *Ohne Phosphor kein gedanke*, exclaims the German materialist. Insanity is disease of the brain-structure, repeats his English brother. It is not so. Phosphorus in sufficient abundance, with a sound molecular structure, is needful and desirable, but brain-soundness and mind-soundness are not one thing, but different things. The brain may be diseased, its structure impaired, without mind-unsoundness. The brain may be most healthy, yet the mental faculties, as regards this terrene life, fled for ever. No, the evil lies quite other than in empirical considerations, resides in the mind's unconsciousness of its consciousness, in a word, the soul's unawareness of its own acts. If in the sane the mind's processes be not objects of attention, unless by a special effort, how much more so then in the insane. During insanity the mind is not conscious of its own consciousness. And herein lies the grand distinction between man and the inferior animals, between sleeping man and waking man, in fine, between man as insane and man as self-conscious. I do not assert that the insane are unconscious, but that they are not conscious of their consciousness, that they do not think on what they are thinking. If they did, and did so healthily, they would cease to be insane. The question of questions in reference to insanity and in respect of the impairment of the principle of volition lies here. I do not say that the man who does not reflect on the processes of his consciousness is insane, but I would assert that the man in insanity does not, indeed, so long as he is insane, cannot do so, and that here lies the point of his disease. The *γνῶθι σεαυτόν* of the ancients, I am persuaded, refers to this, and not merely to the moral consciousness. In short, the insane man does not know himself, and therefore is he insane.

All science, psychology with the rest, consists of a series of approximations to truth. Science never jars with science. There is then no quarrel between physiology and psychology, rightly understood. Each is admirable in its own place. Nay, the phenomena which come within the province of each throw light upon the other, conversely and conversely. But when physiology alone attempts to explain questions that come within the exclusive domain of psychology, what can ensue but error and misconstruction? The questions of criminality and responsibility in insanity, hitherto so obscure, are easily enough resolved, on paper at least, so long as we are careful to keep psychology and physiology in their proper places. If the criminal be conscious of his consciousness, if he be able to reason, combine, in a word, to survey the operations of his inner self, unquestionably he is not insane. But the criminal lunatic, unconscious of his consciousness, and but partially conscious of his acts, is irresponsible because insane. Here, however, there are degrees, for self-consciousness and reason ebb and flow, so that a man may be comparatively sane, therefore responsible at one time, comparatively insane, and therefore irresponsible at another.

Of a surety the bodily health must be looked to in insanity, phosphorus must be furnished, but in copious food-supplies, disordered cerebral action, when it subsists, set aside. But even here, moral treatment is the essential thing. If disease indeed be urgent, if the nerve-structure be lesed, and its function seriously impaired, the man must die. Yet, short of this, the potentiality of recuperation, of self-integration, if I may coin an expression, never wholly intermits, and moral influences come ceaselessly into play. Substitution is the great agent for reclaiming the insane. This, is the moral lever, the mighty engine which is to raise the ruined soul, supplying such allurements as lie within our reach, till at length the principles of self-control and self-assertion being roused, the soul, its nobler powers awakened, gazing face to face on self, is rescued again. Yes, the fixed idea, the revolving circlet of insanity, must be rooted out, not by force or stress of argument, but through a species of gentle yet resistless constraint, until, the work being consummated, the soul become conscious of its better self. Spiritual health, that is what is needed, that is what we must substitute for folly, disease, and, when it subsists, crime. For God has imparted to every man, being cultivated, the divine power of introspection, the faculty of being, doing, thinking well, in a word, of remaining sane. There must be a surcease of all raving, random, circular thought, the soul must be led to higher perceptions, a more purposeful exercise of thought, and man through the providence of man made whole. For insane associations propagate themselves, and therefore must sane associations, at whatever cost of toil and pains, be made to replace them. Use must take the place of disuse in respect of every faculty. The moral decay that neglect and want of care have entailed, must give way to better types of thought and feeling. The will must be disciplined until perfect freedom, the freedom that consists in discharging the divine purpose, reign within. For each successful effort lends fresh powers, self-control breeds self-control, and glorious reason, celestial ray, is at last redeemed.

In nothing are the humaner tendencies of the age more conspicuous than in the general treatment of the insane. Establishments, conductors, as contrasted with former days, vie with each other in avoiding undue restraint, the solace of moral, the mitigation of physical suffering. It must be conceded, however, that the treatment is still too passive, that enough is not yet done to remedy psychical disease. Habitual skill, a practised humanity, will always more or less directly realize their aim, but the best treatment must repose on just views as to the nature of disease. No, insanity does not reside in the absence of phosphorus, the alteration of cerebral tissue, but in the aggravation to extremity of the inanities, follies, crazes, shortcomings of daily life. If a preconceived and most erroneous hypothesis did not blind the judgment, it would be admitted that lunatics at any time might be seen in the enjoyment of perfect bodily health, and without the slightest trace of nervous derangement or cerebral disease whatever. The most successful, and therefore the most rational treatment of lunacy, must involve right views as to its nature and origin. To look upon the malady as material only, is to fly in the face of all observation, all just induction and analysis. As the causes of insanity are moral causes mainly, so must the treatment, the insanity regarded, be a moral treatment mainly. I would rather witness the treatment, the moral treatment of insanity, by a practical, experienced, intelligent non-medical person, than by a medical man entertaining, and only influenced by, materialistic views. For what possible weight can moral treatment, I mean a sufficient moral treatment, have in the eyes of one with whom derangement is a mere psychical question of plus or minus phosphorus, the more or less diseased modification of the molecules of the brain?

The great intelligence and humanity of very many of those who have to do with the treatment considered, I do not think that those who undertake the difficult task of dealing with the insane are afforded sufficient scope. For every means should be wielded calculated to remedy psychical derangement, in short, to reform and integrate, when disordered, the nobler machinery of the soul. More attractive bodily occupation there should be, for one thing, at one time beneath the free heaven, at another in some cheerful, roomy, well-ventilated space indoors. A higher class of persons, better educated, better remunerated, should be entrusted with the immediate culture, so to speak, of the insane. Such would prove susceptible of a far more elevated order of motives than the common herd of keepers and keeperesses, and would correspondingly bring such motives into influential operation in dealing with the insane. Indeed, the latter should be held to constant wholesome occupation of body and soul as free from violence and physical constraint. For it is difficult to imagine the extreme torpor of mind and body into which so many of the insane are plunged. Yet even they, for the most part, might be reached through the medium of their animal wants, various food and clothing, and occupation and recreation, some innocent lingering addiction, which it would be the business of the skilled attendant to discover. To music's gentle solace very many are accessible, and why not, since the musical faculty itself is not insane? When we come to the higher motives furnished by religion, science, letters, art, we find that many are immediately susceptible, and others prospectively so.

For they all help to turn attention from the mental craze, aid our attempts, in individual after individual, to induce sequential effort, the exercise of a more healthy will. Idleness, inoccupation, and gloom are indeed the bane of asylums, where moral culture should come more fully into play. And since the affections and feelings are not necessarily degraded, not even insane, there is in them a perfect mine of moral influence for thoughtful loving intelligence to turn to account when it will. For let us reflect that of the mind in itself we know nothing, know it only in its manifestations, which in the insane are at fault. It is our business, then, to remedy impaired morbid thought, to avert mental ruin, in short, if we can, to rehabilitate into healthy life and action the weakened consciousness of man.

If insanity can be removed, if idiots even, in whom the organs of relation are so deficient, for here lies their entire disease, can be raised to comparative intelligence, elevation, and happiness, with what vastly greater certainty might both these deplorable degradations of humanity be abated. It were indeed a noble problem for the legislator, the moralist, the physician, the divine, to stay altogether these dreary efflorescences of our partial civilization, our false refinement, our imperfectly-cultivated reasoning powers, our neglected youth, our excesses. The realization of a superior social status, of proper habits, would go far to stay the ravages of idiotcy and insanity for ever. For God imparts to man the power of self-conservancy, of maintaining a whole soul. To him has that great Being confided the gift of self-control, and through self-discipline, of guarding against insanity and idiotcy, of realizing success, and purpose, and wit, and strength of healthy will. Few, if any, grow instantly insane. There is many a rally ere the shattered faculties become clouded, impaired, lost. The purposeless will does not all at once forswear its dominancy, but once having lost the mastery is with difficulty restored. For if reason vacate its seat, how is folly to regain it? If indeed we take into account the so frequent weakness and inanity of daily life, we shall only feel surprise that the vacillating will does not yet oftener forsake its throne. Were the great principle of self-control incultivable, insanity could not be prevented, neither could it be removed. For the key to the successful treatment of insanity, and of all tendency thereto, is the substitution of sound for unsound thought, the culture of the God-like faculty of self-control. Any lapse of self-discipline, of self-culture, lessens by so much the practicability, impairs by so much the faculty of self-culture itself. If, in truth, we forego the exercise of our powers, if we forfeit the control which God has confided to us, the more enfeebled do those powers become, the less fitted for healthy life and effort. Lapse breeds lapse, and failure failure, until at length we prove incapable of self-guidance, and come entirely to depend on the providence of our fellows. The insane man sinks into childishness, or a condition approximate, and, like the child, needs firm and succouring care. Mental imbecility, psychical disorder, acknowledge no other source than nature's violated laws. For God requires of us the fulfilment of his purposes, the realization of every obligation; in short, the carrying out of the compact which is entered into with us at our birth. He has gifted us with two worlds, one material, the other immaterial, one somatic, the other psychical, for our heritage. These worlds has he given us, worlds subjected to unerring law, that we should cultivate them and make use of them, and through our conduct beautify them, with, indeed, the dread alternative, in the event of failure, of being stricken with incapacity, and unable to comply with his divinest will.

---

